# Wrapping the stump of the gastroduodenal artery using the ligamentum teres hepatis during laparoscopic pancreaticoduodenectomy: a center’s preliminary experience

**DOI:** 10.1186/s12893-021-01076-8

**Published:** 2021-02-02

**Authors:** Lingwei Meng, He Cai, Yunqiang Cai, Yongbin Li, Bing Peng

**Affiliations:** Department of Pancreatic Surgery, West China Hospital of Sichuan University, No. 37, Guoxue Alley, Chengdu, 610041 Sichuan China

**Keywords:** Postpancreatectomy hemorrhage, Laparoscopic pancreaticoduodenectomy, Ligamentum teres hepatis, Gastroduodenal artery stump

## Abstract

**Background:**

The present study aims to assess the preliminary outcomes of the effectiveness of wrapping the ligamentum teres hepatis (LTH) around the gastroduodenal artery stump for the prevention of erosion hemorrhage after laparoscopic pancreaticoduodenectomy (LPD).

**Methods:**

We reviewed 247 patients who had undergone LPD between January 2016 and April 2019. The patients were divided into two groups according to whether LTH wrapped the stump of the gastroduodenal artery: group A (119 patients) who underwent the LTH wrapping procedure, and group B (128 patients) who did not undergo the procedure. The perioperative data from the two groups were reviewed to assess the effectiveness of the LTH procedure for the prevention of postpancreatectomy hemorrhage (PPH) and other complications.

**Results:**

No differences were observed in the clinical characteristics between the two groups. The data from 247 patients were acceptable for analysis: 119 patients underwent wrapping, and 128 patients did not. The incidence of clinically relevant pancreatic fistula (8.4% vs 3.9%), biliary fistula (2.5% vs 1.6%), intra-abdominal infection (10.1% vs 3.9%) and delayed gastric emptying (13.4% vs 16.4%) showed no significant difference between group A and group B. The 90-day mortality and 90-day reoperation rates (0.8% vs 0.8% and 5.0% vs 3.1%) were also similar between group A and group B. Furthermore, postpancreatectomy hemorrhage of Grade B and C occurred in 0 patients (0.0%) in the wrapping group, which was significantly less frequent than the occurrence in the nonwrapping group (7 patients; 5.5%, P = 0.02).

**Conclusions:**

Wrapping the LTH around the gastroduodenal artery stump after LPD does not reduce the incidence of clinically relevant pancreatic fistula, biliary fistula or delayed gastric emptying. However, this procedure has a trend of reducing the rate of PPH of Grade B and C after LPD and is simple to perform.

## Background

Pancreatic fistula remains one of the most harmful and troublesome complications after laparoscopic pancreaticoduodenectomy (LPD) [1–3]. It remains the single determinant of main postoperative morbidity and mortality related to pancreatic resection and plays a vital role in terms of operation-related mortality, morbidity, length of postpancreatectomy stay, and economic impact [4, 5]. In addition, it may cause many other serious complications, of which postpancreatectomy hemorrhage (PPH) may be fatal. Pancreatic fistula may directly expose skeletonized or divided vessels, especially a gastroduodenal artery (GDA) stump, to active pancreatic juice, forming a region that may result in vessel erosion or even delayed PPH. With the aim of protecting vessels near the pancreatic stump from potential pancreatic fistulas, we have adopted a surgical option by which these vessels are wrapped using the ligamentum teres hepatis (LTH) which has been described previously [6].

In pancreatic surgery, the LTH has been used to prevent the formation of pancreatic fistula or a falciform ligament flap for the protection of the gastroduodenal artery stump after pancreaticoduodenectomy [7–9]. However, the methods described above all used open surgery, in this case–controlled study, we further describe the experience of using the LTH to protect the gastroduodenal artery (GDA) stump during LPD to prevent PPH.

## Methods

This is a retrospective observational study. Our study included all patients who underwent LPD from January 2016 to April 2019. We routinely use the LTH to wrap around the GDA stump after March 2018 while never done before. All clinical, biochemical, and radiological data were collected retrospectively from our own center database. The specific parameters of patients analyzed included the incidence of PPH, clinically relevant pancreatic fistula, intra-abdominal abscess, delayed gastric emptying, length of operating time and postoperative hospital stay, and the interventions to treat complications and their outcomes.

### Perioperative data collection

The preoperative data comprised information on age, sex, body mass index (BMI), American Society of Anesthesiologists (ASA) score and presence of comorbidity. The intraoperative data collected from both groups included the length of operative time, blood loss, perioperative blood transfusion, the diameter of pancreatic duct, and pancreatic texture. The postoperative data included postoperative complications (the clinically relevant pancreatic fistula, biliary fistula, PPH and delayed gastric emptying), 90-day mortality, and 90-day reoperation. All the patients were thoroughly informed about the procedure, risks, and potential advantages of the method. Written informed consent was obtained from all the patients in our study, which was approved by the ethics committee of Sichuan University.

### Surgical techniques for mobilization of the LTH and wrapping of the GDA stump

**Fig. 1.**
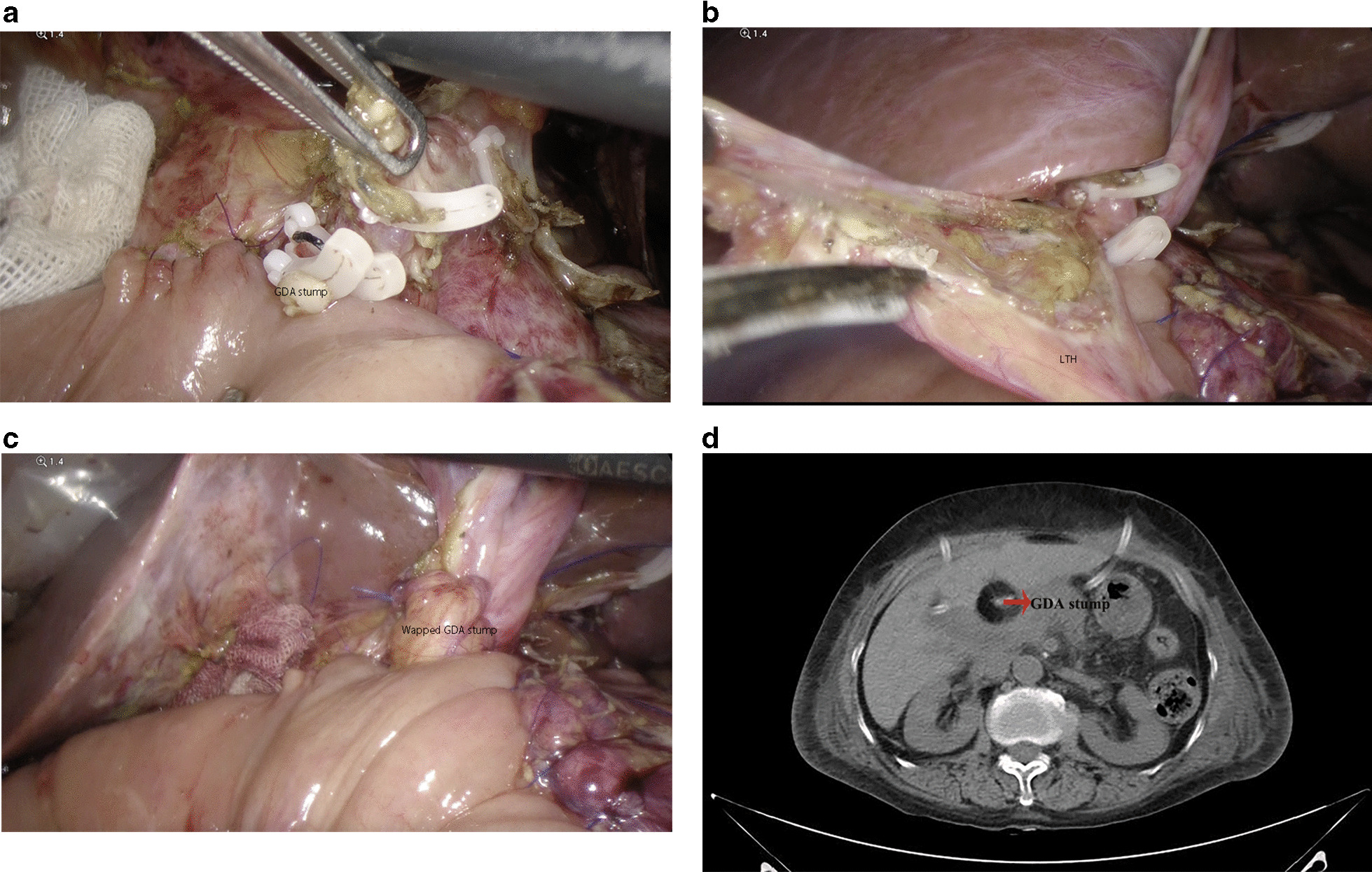
**a** The LTH is detached from the abdominal wall from the umbilicus to the liver. **b** After the anastomosis, the LTH is divided, extended used as a vascular pedicle. **c** The LTH is used to cover the GDA stump. **d** Postoperative CT scan showing the wrapped GDA stump after a LPD procedure

The GDA stump was exposed in the visual field (Fig. 1a). The LTH (Fig. 1b) was mobilized by dividing it around the GDA stump. The fat near the ligament was preserved. The vessels between the ligament and liver parenchyma were ligated and divided. Using this method, we achieved a flap length of approximately 10 cm. The GDA stump was routinely fixed with 4–0 or 3–0 polypropylene sutures (Fig. 1c). The postoperative CT scan revealed a thick fat-density area, corresponding to the LTH, that completely wrapped a well-enhanced vessel, the GDA stump. The drain was placed on the superior edge of the area (Fig. 1d).

### Definitions

Morbidities within 90 postoperative days were stratified by the Clavien–Dindo classification of surgical complications [10]. Postoperative pancreatic fistula (POPF), delayed gastric emptying (DGE), chylous leakage and postpancreatectomy hemorrhage (PPH) were defined according to the International Study Group [11–13]. Reoperation was defined as a secondary operation due to severe complications within 90 days following LPD. The patients were discharged when oral intake and moderate activity were tolerated without any abnormal postoperative complications or laboratory findings.

### Statistical analysis

Data for continuous variables were expressed as the mean ± standard deviation and compared with data from a normal distribution using Student's t test. Categorical variables were compared using the Chi-square test or Fisher's exact test as appropriate. Statistical analysis was performed using SPSS version 23.0 (SPSS Inc., Chicago, IL, USA). P < 0.05 was considered statistically significant.

## Results

For all patients, there were at least 90 days of follow-up, allowing ample time for surgical complications to become apparent. No patients were lost to follow-up. We successfully wrapped the stump of the GDA in 119 patients who underwent LPD after March 2018, and it took approximately 10–15 min to perform the wrapping. Postoperative CT examinations revealed an unenhanced low-density area corresponding to the wrapped LTH surrounding the GDA stump (Fig. 1d).

The patients’ parameters of the two groups included age, male/female ratio, BMI and ASA score. These data showed no statistical significance (Table 1).

**Table 1:** Variables of the two groups

Variables	Group A (n = 119)	Group B (n = 128)	*P* value
Age (years, mean ± SD)	60.0 ± 13.1	59.6 ± 12.3	0.85
Male/female (n)	63/56	78/50	0.21
BMI (kg/m^2^, mean ± SD)	22.22 ± 2.79	22.17 ± 2.88	0.90
ASA score (*n*, %)			
II	96(80.7%)	93 (72.6%)	0.14
III	22 (18.5%)	34 (26.6%)	0.13
IV	1 (0.8%)	1 (0.8%)	> 0.99

*BMI* body mass index, *ASA* American Society of Anesthesiologists

Relevant data about the health status of the patients included information about comorbidities, history of abdominal surgery, intraoperative blood loss and transfusion, pancreas texture, pancreatic duct and tumor size and biochemical estimations. Group-wise analysis revealed no statistical significance between the two groups (Tables 2, 3).

**Table 2:** Variables of the two groups before operation

Variables	Group A (n = 119)	Group B (n = 128)	*P* value
Comorbidities			
COPD (*n*, %)	9 (7.6%)	7 (5.5%)	0.50
Chronic gastritis or duodenitis (*n*, %)	3 (2.5%)	5 (3.9%)	0.72
Hypertension (*n*, %)	27 (22.7%)	28 (21.9%)	> 0.99
Diabetes (*n*, %)	15 (12.6%)	21 (16.4%)	0.40
CVD (*n*, %)	6 (5.0%)	8 (6.3%)	0.68
Hemoglobin (g/dL, median, IQR)	122 (109–133)	121 (108–137)	0.83
Serum bilirubin (mg/L, median, IQR)	34 (12–142)	26 (11–146)	0.93
ALT (IU, median, IQR)	50 (18–128)	32 (15–100)	0.40
Serum albumin (g/L, median, IQR)	39 (35–43)	38 (33–41)	0.12
White blood cell count (n × 10^9^/L, median, IQR)	5.6 (4.5–6.9)	5.5 (4.5–6.7)	0.81
Tumor location			
DCBD (n, %)	18 (15.1%)	27 (21.1%)	0.23
Pancreas (n, %)	67 (56.3%)	64 (50.0%)	0.32
Ampulla (n, %)	11 (9.3%)	6 (4.7%)	0.16
Duodenum (n, %)	23 (19.3%)	31 (24.2%)	0.35

*COPD* chronic obstructive pulmonary disease, *CVD* cardiovascular disease, *ALT* alanine aminotransferase, *DCBD* distal common bile duct, *IQR* interquartile range

**Table 3:** Intraoperative variables of the two groups

Variables	Group A (n = 119)	Group B (n = 128)	*P* value
Duration of surgery (min, mean ± SD)	318.3 ± 75.3	374.7 ± 86.3	< 0.001
Operative blood loss (mL, mean ± SD)	144.3 ± 109.4	161.3 ± 174.5	0.37
Perioperative blood transfused (n, %)	9 (7.6%)	15 (11.7%)	0.27
Pancreas texture (*n*)			
Soft/firm	37/82	43/85	0.68
Pancreatic duct diameter (*n*, %)			
≤ 3 mm	100 (84.0%)	102 (79.7%)	0.38
> 3 mm	19 (16.0%)	26(20.3%)	
Tumor size (cm, mean ± SD)	3.0 ± 1.5	2.7 ± 1.7	0.79

Postoperatively, 7 patients (5.5%) developed PPH of grade B and C in group B, while 0 patients in group A showed significant differences (P = 0.02, Table 4). Three patients developed potentially significant hemorrhage associated with clinically relevant pancreatic fistula and further developed intraabdominal bleeding from the stump of the GDA and hepatic artery (HA). The pseudoaneurysm of left hepatic artery and GDA stump was successfully embolized through interventional surgery in these patients. We analyzed the data and found that chylous leakage of grade B occurred more frequently in group A than in group B (P = 0.04), resulting in a longer time to remove all drainage tubes in group A than in group B. Other complications included delayed gastric emptying no matter the grade, intra-abdominal infection, bile leakage and the Clavien–Dindo classification greater than level 3 showed no statistical significance. The mean postoperative hospital stay was 17.2 days in group A, which longer than in group B (P = 0.04) may be associated with the higher rate of chylous leakage. No patients died due to the clinically relevant pancreatic fistula in the two groups. No differences were observed in 90-day mortality, 90-day reoperation, and 90-day reoperation related to PPH between the two groups. However, time to first passage of flatus and time to first oral intake showing obvious differences may be related to the application of enhanced recovery after surgery in pancreatic surgery.

**Table 4:** Postoperative conditions of the two groups

Conditions	Group A (n = 119)	Group B (n = 128)	*P* value
Clinically relevant pancreatic fistula	10 (8.4%)	5 (3.9%)	0.18
Grade B	7 (5.9%)	5 (3.9%)	0.56
Grade C	3 (2.5%)	0 (0.0%)	0.11
Post-pancreatectomy hemorrhage (PPH)	4 (3.4%)	8 (6.3%)	0.38
Grade A	4 (3.4%)	1 (0.8%)	0.20
Grade B	0 (0.0%)	4 (3.1%)	0.12
Grade C	0 (0.0%)	3 (2.3%)	0.25
Grade B + C	0 (0.0%)	7 (5.5%)	0.02
Chylous leakage	19 (16.0%)	11 (8.6%)	0.08
Grade A	5 (4.2%)	5 (3.9%)	> 0.99
Grade B	14 (11.8%)	6 (4.7%)	0.04
Grade C	0 (0.0%)	0 (0.0%)	NS
Delayed gastric emptying	16(13.4%)	21(16.4%)	0.52
Grade A	6 (5.0%)	11(8.6%)	0.27
Grade B	6 (5.0%)	8 (6.3%)	0.68
Grade C	4 (3.4%)	2 (1.6%)	0.43
Intra-abdominal infection	12(10.1%)	5 (3.9%)	0.08
Biliary fistula	3 (2.5%)	2 (1.6%)	0.67
Input loop obstruction	5 (4.2%)	1 (0.8%)	0.11
Incision infection	2 (1.7%)	2 (1.6%)	> 0.99
Clavien–Dindo ≥ III	14(11.8%)	7 (5.5%)	0.08
Mortality due to pancreatic fistula	0 (0.0%)	0 (0.0%)	NS
90-day mortality	1 (0.8%)	1 (0.8%)	> 0.99
90-day re-operation	6 (5.0%)	4 (3.1%)	0.53
90-day re-operation relate to PPH	0 (0.0%)	3 (2.3%)	0.25
Postoperative hospital stay (days)	17.2 ± 8.3	15.3 ± 6.2	0.04
Time to first passage of flatus (days)	2.9 ± 0.8	3.3 ± 1.0	< 0.001
Time to remove all drainage tube(days)	12.5 ± 7.8	9.3 ± 4.5	< 0.001
Time to first oral intake (days)	2.5 ± 1.6	3.6 ± 2.7	< 0.001

## Discussion

The overall incidence of clinically relevant pancreatic fistulas at our center is 6.1%. The overall rate of post-pancreatectomy hemorrhage is 4.9%, and hemorrhage occurred at three patients (20%) with clinically relevant pancreatic fistulas. The incidence of massive hemorrhage was reported in patients with pancreatic fistulas to be 16–40% [14, 15]. Therefore, hemorrhage may still occur even after the stump of the GDA has been wrapped, although the incidence of hemorrhage in this study was lower than the reported incidence, and only one died due to PPH from GDA stump in the nonwrapping group. Although there is no statistical difference in 90-day re-operation relate to PPH, it still shows a tendency to reduce the rate. The successful results may be related to the novel surgical technique, including the use of surgeon’s skilled Bing's pancreatojejunostomies [16], the correct placement of the drainage tube, timely diagnosis of pancreatic fistula using CT scan, and opportune invasive intervention. In addition, the simple wrapping technique that was used may work to the prevention of hemorrhage.

The prevention of PPH is also a major concern in LPD. The falciform ligament and omental flaps have been used to pack the stump of the GDA during pancreaticoduodenectomy [17–21]. Shah et al. [19] and Seyama et al. [22] revealed that omental wrapping can significantly reduce postoperative complications, such as post-pancreatectomy hemorrhage, delayed gastric emptying, and biliary fistula. Omental wrapping can decrease the occurrence of pancreatic fistula and protect skeletal vessels to prevent PPH. However, data from other studies have shown different conclusions [23]. To the best of our knowledge, use of the LTH to cover the stump of the GDA stump during LPD surgery has only been described by our center as video article [6]. From our experience, the surgical technique, which includes ligamentum teres hepatis flap preparation, mobilization, and suprascapular suturing, can be easily standardized and does not relevantly prolong operation time (Table 2). Our study revealed that the LTH significantly reduced postoperative complications, such as postpancreatectomy hemorrhage of grade B and C, while it did not decrease the rate of clinically relevant pancreatic fistula, biliary fistula, intra-abdominal infection or delayed gastric emptying. LTH wrapping did not significantly reduce the incidence of pancreatic fistula. Similar findings were reported by Tani et al. [23], who found that omentum ligament wrapping did not decrease the incidence of pancreatic fistula.

The presented surgical procedure is a simple and easy technique for the separation of skeletal GDA stump from the area of pancreaticoduodenectomy. It is suggested that the LTH can prevent the diffusion of pancreatic juice with or without bacterial infection and can protect the skeletal GDA stump and hepatic artery. In this study, no patients who underwent the LTH wrapping procedure developed PPH of grade B or C. However, it could not be confirmed whether the present surgical option itself prevented PPH. Further controlled randomized studies involving large numbers of patients are necessary to confirm the value of the present technique in LPD.

## Conclusion

In conclusion, one retrospective single-center data can hardly provide sufficient evidence for the use of LTH wrapping because of the relatively low incidence of erosion hemorrhage after LPD. The present study pooled our center’s currently available data on this issue. Despite the low occurrence of PPH after LPD, the significant reduction in the incidence of PPH of grade B and C with LTH wrapping marks a major step toward safer LPD surgery because of its high mortality. The wrapping of GDA stump using the LTH is technically easy, and our study found this procedure may have a role of decreasing PPH caused by clinically relevant pancreatic fistula following LPD. However, a large sample and prospective randomized trials are still needed to verify this conclusion.

## Data Availability

The data and materials used and/or analyzed during the current study are available from the corresponding author on reasonable request.

## Abbreviations

LTH: Ligamentum teres hepatis
LPD: Laparoscopic pancreaticoduodenectomy
PPH: Postpancreatectomy hemorrhage
GDA: Gastroduodenal artery
BMI: Body mass index
ASA: American Society of Anesthesiologists
